# Physical Health and Quality of Life among Older People in the Context of Chinese Culture

**DOI:** 10.3390/ijerph18136798

**Published:** 2021-06-24

**Authors:** Lin Zhang, Xinjie Wei, Xueyao Ma, Zhihong Ren

**Affiliations:** 1 School of Psychology, Central China Normal University, Wuhan 430056, China; lin_zhang@ccnu.edu.cn (L.Z.); tillywei@mails.ccnu.edu.cn (X.W.); 2 Key Laboratory of Adolescent Cyberpsychology and Behavior, Ministry of Education, Wuhan 430056, China; 3 Key Laboratory of Human Development and Mental Health of Hubei Province, Wuhan 430056, China; 4 Department of Psychosomatic Medicine and Psychotherapy, University of Ulm, Medical Psychology, Frauensteige 6, 89075 Ulm, Germany; xueyao_ma@hotmail.com

**Keywords:** physical health, quality of life, positive cognition, negative emotion, serial mediation, older people, Chinese culture

## Abstract

Population aging has become a crucial problem in China. Recently, the Chinese government has adopted many strategies and policies to solve this problem and improve the quality of life of older individuals. The present study aimed to examine the effect of physical health on quality of life among older individuals in the context of Chinese culture and explore the potential mediating roles of positive cognition and negative emotions in the association between physical health and quality of life. Data were from the wave of 2017–2018 of the Chinese Longitudinal Healthy Longevity Survey. Data on physical health, quality of life, positive cognition, and negative emotions of 15,874 older people were included in the present study. Pathway analysis was conducted by using IBM SPSS AMOS 21.0, and double verified using PROCESS Macro for SPSS 3.5.3. Results showed that physical health was positively associated with quality of life among older individuals in the context of Chinese culture. The effect size was small to moderate. Positive cognition and negative emotions independently and serially mediated the linkage of physical health and quality of life. These findings provided empirical evidence for the activating event-belief-consequence theory of emotion and hierarchy of needs theory and indicated that Chinese older people focused more on physical health rather than mental health. Practitioners could teach older individuals strategies of emotion regulation and cognitive appraisal to improve the quality of life of older individuals.

## 1. Introduction

Rapid aging is becoming an important public health issue in the global population. Problems associated with aging have become serious in China as well. As of 2020, China has approximately 180 million older people aged 65 years or older, which account for 13% of the population [1]. The Chinese government has adopted a series of actions to address this challenge, including raising the retirement age and allowing all couples to have three children. Improving the quality of life and life satisfaction of older individuals in China is important with the advent of an aging society. Therefore, it is of great practical significance to explore the influential factors of quality of life and life satisfaction among older individuals in China.

Physical health is a low-level and basic need for all individuals according to Maslow’s hierarchy of needs theory [2]. However, older individuals present a large number of physical features that are different from their younger counterparts, including a deterioration of physical capacities and recession of body function [3,4]. That is, older people may have to face the fact that their basic needs may not be satisfied. When a low-level and basic need cannot be satisfied, it is difficult for older individuals to pursue a higher level of need and experience negative feelings [5]. Therefore, physical health would affect their quality of life and life satisfaction. A number of previous studies have confirmed this argument. For example, a systematic review and meta-analysis found that frailty was associated with worse quality of life among community-dwelling older people, which was strong evidence for the argument [6].

The concept of quality of life and life satisfaction differs across cultures; therefore, it is crucial to understand this concept in the cultural context. For example, compared with collectivistic cultures, life satisfaction is more likely to be affected by hedonic balance for people living in individualistic cultures [7]. Western people value mental health in life satisfaction more than Eastern people who live in collectivistic cultures [8]. A previous study found that older Japanese adults considered that functional fitness would affect their life satisfaction [9]. In the context of Chinese culture, frequent interaction with family members, some unique Chinese cultural beliefs (e.g., beliefs about adversity, “mianzi”) were considered to be associated with life satisfaction among older people [10,11]. Mianzi (face) is others’ recognition of an individual’s social status [12]. Therefore, it is necessary to examine the effect of physical health on quality of life/life satisfaction in the context of Chinese culture.

Another important question is how physical health affects the quality of life/life satisfaction among older people in China. Indirect effects (i.e., mediators in the linkage of physical health and quality of life) need to be examined. Ellis believes that an activating event (i.e., physical status) is an indirect cause of the emotional and behavioral consequence (quality of life/life satisfaction), while the direct cause of consequence is the belief that arises from the individual’s perception and evaluation of the activating event [13]. It is necessary to examine whether cognition/belief and emotion mediate the linkage of physical health and quality of life/life satisfaction.

In the present study, we examine the effect of physical health on quality of life/life satisfaction among older people in the context of Chinese culture. In addition, the pathway from physical health and quality of life/life satisfaction (i.e., mediating variables) is also be examined.

### 1.1. Theoretical Bases

The theoretical bases of the present study include the hierarchy of needs theory and the ABC theory of emotion [2,13]. The ABC Theory of Emotion was proposed by Ellis in the 1950s [13]. He considered that individuals’ moods and behavior (i.e., C: consequence) were not caused by activating events (i.e., A: activating event) but caused by the interpretation and evaluation of the event (i.e., B: belief) [13].

The hierarchy of needs theory, a five-stage hierarchy of human needs, was proposed by Maslow in 1943, in which physiological needs and safety belong to basic and low-level needs, which have to be satisfied first before pursuing higher-level needs [14].

### 1.2. Physical Health and Quality of Life

According to the hierarchy of needs theory, physical health is a basic need of all persons [2,14]. Only once this basic requirement is met, can older individuals feel the satisfaction of life and pursue higher-level needs. Kojima et al. conducted a systematic review and meta-analysis to examine the relationships between frailty and quality of life among community-dwelling older people and found a consistent inverse association between them [6]. They suggested that interventions aiming at reducing frailty are helpful to improve the quality of life for older individuals [6]. Some researchers also found that physical exercise can help improve quality of life in an intervention study [15]. Above all, it is reasonable to hypothesize that physical health would affect the quality of life among older individuals.

However, the extent to which physical health affects the quality of life among older individuals in the context of Chinese culture needs to be studied more extensively. Regarding the influential factors of quality of life/life satisfaction, most of the previous studies were conducted in developed countries. A longitudinal study conducted in the U.S. found that the effect of physical health on quality of life was limited and indicated that psychological well-being might be more important than physical health for older individuals living in the U.S. [16]. A population-based observational study showed similar results: physical performance was significantly but weakly associated with life satisfaction, while mental health was strongly associated with life satisfaction among older individuals in the Netherlands [17].

However, to the best of our knowledge, a limited number of studies have explored the importance of physical health in the quality of life among older individuals in Eastern cultures. A previous study found that older individuals in Taiwan valued physical health the most when they were asked the question on the essential components of an ideal and satisfactory life in old age [18]. An investigation in 2011–2012 found that physical health and economic status were the most predictive of life satisfaction for older people aged no less than 80 years old in China, but this study was conducted ten years ago [19]. Overall, compared with Western cultures, physical health seems more important for older people living in Eastern cultures.

Therefore, considering the differences in the importance of physical health on quality of life in diverse cultural contexts, it is necessary to examine the effect of physical health on the quality of life in the context of Chinese culture. We hypothesized that the effect size of physical health on quality of life among older individuals in the context of Chinese culture would be moderate.

### 1.3. The Mediating Role of Positive Cognition

Physical status may affect human cognition [20]. When an individual is suffering from physical diseases, she or he is more likely to have negative thoughts [21]. These negative thoughts may lead to a decrease in quality of life [22]. A cross-sectional study found that irrational beliefs and attitudes were negatively associated with quality of life. More specifically, irrational beliefs explained approximately 9% and attitude to death explained about 30% of the variance in quality of life among older adults [23]. Another cross-sectional study also found that if older individuals were optimistic about their control ability for the environment, they would achieve greater life satisfaction [24]. The negative thoughts can be modified and positive cognition can be developed through psychological interventions, such as cognitive-behavior treatment [25].

### 1.4. The Mediating Role of Negative Emotions

Physical health has an influence on negative emotions. More precisely, a number of previous longitudinal studies found that there was a reciprocal relationship between physical health and negative emotions [26,27]. A consensus has been reached about this relationship [26]. Moreover, negative emotions may affect the quality of life/life satisfaction of older individuals. Many previous studies found that negative moods and emotions related to depression and loneliness can predict the quality of life among older people across countries [17,19,28].

### 1.5. The Serial Mediating Role of Positive Cognition and Negative Emotions

Positive cognition can decrease negative emotions. As claimed by Ellis in his ABC theory of emotion, emotions can be directly triggered by cognition (i.e., the belief, interpretation, and evaluation of activating events) [13]. A Spanish nationwide investigation also found an association between control beliefs and negative effects among older individuals in Spain, which supported the theory [24]. Moreover, a large number of intervention studies also found that learning reappraisal is a key ingredient of many interventions such as cognitive and cognitive-behavioral treatment to decrease negative emotions [29].

### 1.6. The Current Study

Overall, the present study aimed to examine the effect of physical health on quality of life among older individuals in the context of Chinese culture, further explore how physical health affects the quality of life (i.e., the mediating roles of positive cognition and negative emotions in the association between physical health and quality of life). Our hypotheses are as follows:

**Hypothesis** **1** **(H1).** *Physical health may be positively associated with quality of life among older Chinese people.*

**Hypothesis** **2** **(H2).** *Positive cognition may play a mediating role in the association between physical health and quality of life.*

**Hypothesis** **3** **(H3).** *Negative emotion may play a mediating role in the association between physical health and quality of life.*

**Hypothesis** **4** **(H4).** *Positive cognition and negative emotion may play a serial-mediation role in the association between physical health and quality of life.*

## 2. Materials and Methods

### 2.1. Study Population

In the present study, data from 15,874 older people were used from a recent wave (2017–2018) of the Chinese Longitudinal Healthy Longevity Survey (CLHLS), which can be accessed from the Peking University Open Research Data Platform (https://opendata.pku.edu.cn/ accessed on 24 June 2021). As the largest survey of older people in China, the sample of the survey represented approximately 85% of the total Chinese population, covering 23 provinces or autonomous regions across China. The purpose of the survey was to investigate the physical and mental health status of older people aged no less than 65 years old (e.g., social and economic background, family structure, economic source, and economic situation, health, and quality of life). More details about the information of the survey may be seen in Gu et al.’s reports [30].

The CLHLS was approved by the Biomedical Ethics Committee, Peking University. Prior to the start of the survey in each wave, older participants were informed clearly that their participation in the survey on a voluntary and anonymous basis, and they had the right to quit the survey at any time for any reason. All the data were collected in person for research purposes only. All participants gave their written consent.

### 2.2. Measures

In the CLHLS, the questionnaire for interviews of the surviving participants was utilized to measure the physical and mental health status of older people. The reliability, validity, and internal consistency of the questionnaire have been verified in previous studies [30,31]. In the present study, we utilized items that measured physical health, positive cognition, negative emotions, and quality of life in the questionnaire. Items of the questionnaire were reverse scored for ease of understanding. More details were introduced as follows:

#### 2.2.1. Outcome

The outcome (i.e., self-perceived quality of life) was measured using one self-reported item “How do you rate life at present”. Participants were asked to rate it on a 5-point Likert scale (1 = *very good* to 5 = *very bad*). A high score means a high level of self-perceived quality of life.

#### 2.2.2. Independent Variable

Physical health was regarded as the independent variable and measured using one self-reported item “How do you rate your health at present?” on a 5-point Likert scale (1 = *very good* to 5 = *very bad*). A high score means a high level of physical health.

#### 2.2.3. Mediators

One of the mediators (i.e., positive cognition) was measured using one self-reported item “Do you always look on the bright side of things?” Participants were asked to rate this item on a 5-point Likert scale (1 = *always* to 5 = *never*). A high score represents a high level of positive cognition.

Negative emotion was the other potential mediator and measured using the following items “Are you bothered by things that don’t usually bother you?” and “Do you feel sad, blue, or depressed?” A higher total score represents a higher level of negative emotions of older people. The internal consistency for the scale was evaluated by calculating Cronbach’s alpha coefficient. An acceptable internal consistency was reached with a Cronbach’s alpha of 0.728 for the scale.

### 2.3. Data Analysis

Descriptive statistics, correlation analyses, and pathway analyses were conducted using IBM SPSS AMOS 21.0 (IBM Corp, Armonk, NY, USA), and double verified using PROCESS Macro for SPSS 3.5.3 (http://processmacro.org/download.html accessed on 24 June 2021). Specifically, first, descriptive statistics were conducted for all variables and correlation analyses were conducted for all continuous variables. Furthermore, pathway analyses were conducted to examine independent or serial mediating roles of positive cognition and negative emotions in the relationship between physical health and quality of life when controlling for age and gender. We calculated 95% confidence intervals (CIs) using the bias-corrected boot-strapped method based on 10,000 samples to determine the statistical significance of indirect effects.

## 3. Results

### 3.1. Descriptive Statistics and Correlation Analyses

Table 1 shows the results of descriptive statistics. A total of 15,874 older individuals were included in the analysis, including 8949 women and 6925 men, with an average age of 85.46 years. Among them, 6135 participants were married (38.6%), 276 participants were separated (1.7%), 52 participants were divorced (0.3%), 9004 participants were widowed (56.7%), 140 participants were never married (0.9%), 267 (1.8%) participants did not report their marital status.

As shown in Table 2, physical health (r = 0.496, *p* < 0.01) and positive cognition (r = 0.379, *p* < 0.01) were positively correlated with quality of life. Negative emotions (r = −0.278, *p* < 0.01) was negatively correlated with quality of life. Furthermore, physical health was positively correlated with positive cognition (r = 0.370, *p* < 0.01) but negatively correlated with negative emotions (r = −0.308, *p* < 0.01). Positive cognition was negatively correlated with negative emotions (r = −0.409, *p* < 0.01).

### 3.2. Test of Mediation

**Figure 1:**
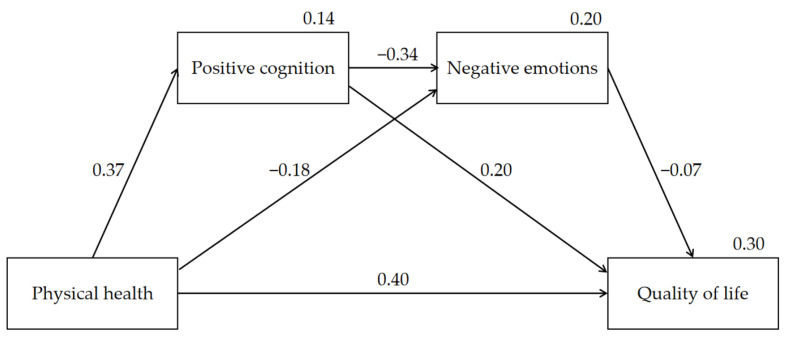
The serial-mediation model showing physical health, positive cognition, and negative emotions on quality of life.

After controlling for age and gender, we examined the serial mediating model by using SPSS AMOS 24.0. The standardized coefficients for total and direct effects on physical health, positive cognition, negative emotions, and quality of life in the serial mediation model are shown in Table 3 and Figure 1. Specifically, physical health had a direct and positive association with both quality of life (standardized path coefficient = 0.400, *p* < 0.001) and positive cognition (standardized path coefficient = 0.370, *p* < 0.001). This physical health had a direct and negative association with negative emotions (standardized path coefficient = −0.182, *p* < 0.001). The positive cognition had a direct and negative association with negative emotions (standardized path coefficient = −0.342, *p* < 0.001), conversely, it had a direct and positive association with quality of life (standardized path coefficient = 0.202, *p* < 0.001). Negative emotions was directly associated with quality of life (standardized path coefficient = −0.072, *p* < 0.001).

The total, individual, and serial indirect effects for physical health on quality of life are shown in Table 4. The significance of indirect effects was tested by the bias-corrected 95% confidence intervals (CIs). That is, if the bias-corrected 95% confidence intervals precluded zero, the indirect effects were significant. Specifically, the indirect effect of physical health on quality of life via a pathway of positive cognition was significant (indirect effect = 0.075, 95% CI = 0.067–0.082). The effect of physical health on quality of life was also mediated by negative emotions (indirect effect = 0.013, 95% CI = 0.010–0.016). Furthermore, a significant indirect effect on the quality of life via positive cognition and negative emotions was found for physical health (indirect effect = 0.009, 95% CI = 0.007–0.011). Overall, the total effect of physical health on quality of life was 0.497, of which 80.5% (0.400) was direct and 19.5% (0.097) was indirect.

## 4. Discussion

In the present study, we investigated the effect of physical health on self-perceived quality of life among a very large sample of Chinese older individuals and examined the potential pathway between physical health and self-perceived quality of life, that is, the mediating roles of positive cognition and negative emotions in the linkage of physical health and self-perceived quality of life. With the onset of an aging society in China, the quality of life of older people has become an important concern not only for older individuals themselves but also for Chinese society. Older people have to contend with the fact that their physical health declines with aging. Therefore, it is necessary to investigate how and to what extent physical health affects the quality of life among Chinese older people. In the present study, we found that physical health was positively associated with self-perceived quality of life among Chinese older people. Moreover, positive cognition and negative emotions independently and serially mediated the association between physical health and self-perceived quality of life.

The strengths of the present study may be summarized as follows: first, one of the strengths of the present study was that the sample of this study was representative of typical older adults in China. Data in the present study were selected from the recent wave (2017–2018) of the Chinese Longitudinal Healthy Longevity Survey (CLHLS), which was the largest survey for investigating the physical and mental status among older people in China and collected data of 15874 older individuals in the 2017–2018 wave. Second, the present study contributed to our understanding of the perceived quality of life of older individuals. The 36 short-form health survey (SF-36) was used to measure the quality of life of Chinese older adults in many previous studies [32]. In the present study, a direct measurement of assessing the self-perceived quality of life was utilized (i.e., older individuals were asked to rate their perceived quality of life directly). Third, the present study examined the effect of physical health on self-perceived quality of life among older people in the context of Chinese culture. Undoubtedly, physical health is an important factor in the quality of life across cultures. However, the extent to which physical health affects the quality of life may vary among different cultures [33]. Therefore, it is necessary to investigate how and to what extent physical health affects the self-perceived quality of life in the context of Chinese culture.

There were some encouraging findings in the present study. A positive association between physical health and self-perceived quality of life was found among Chinese older people, which suggested that a physically healthier older individual might perceive a higher quality of life. The effect size was small to moderate, however. We also found that this association was mediated both independently and serially by positive cognition and negative emotions in the present study.

Specifically, first, we found that physical health had a positive effect on self-perceived quality of life among older people, which was consistent with previous studies [17,34]. We also found that the effect sizes were different across cultures. For example, a population-based observational study found that older people in the Netherlands considered that their life satisfaction was more affected by mental health compared with physical health [17]. On the contrary, a previous survey in Taiwan found that older individuals in Taiwan most value physical health in their aging life [18]. In the present study, we found that the effect size of physical health on self-perceived quality of life was small to moderate, which indicated that physical health is a small part of older adults’ aging life in China. Other factors (e.g., relationships between older individuals and their offspring, their relationships with other older people in the community) may also contribute to the quality of life in the context of Chinese culture because the Chinese pay much attention to relationships and China has a cultural background of raising children to care for parents in their old age [35].

Moreover, in the present study, we found that positive cognition mediated the association between physical health and self-perceived quality of life as we hypothesized. This result provided limited evidence for the ABC theory proposed by Ellis [13]. In that theory, Ellis claimed that cognition mediated the linkage of the objective event (i.e., physical health) and consequence (i.e., self-perceived quality of life). Except for positive cognition, our results showed that negative emotions also mediated the association between physical health and self-perceived quality of life. A good physical condition may decrease older individuals’ negative emotions. The decrease in negative emotions may contribute to increasing the self-perceived quality among older people according to the emotion theories of James-Lange and Cannon [36]. Furthermore, we found that the indirect effect from physical health to quality of life through positive cognition was larger than the indirect effect through negative emotions, which indicated that positive cognition may be a more important mediator in the linkage of physical health and self-perceived quality of life for older individuals. A previous study also found that older people were more likely to use cognitive reappraisal and less likely to use suppression to regulate their emotions, which was consistent with our results [37].

Lastly, we found that positive emotion and negative emotions serially mediated the association between physical health and self-perceived quality of life as we hypothesized. This result provides strong empirical support for the ABC theory of emotion proposed by Ellis [13]. Ellis considered that the emotions of individuals were not elicited by events directly in their lives, but induced by the interpretations/beliefs/cognitions we made of those events [13].

## 5. Implications and Limitations

These findings may have several important implications in general. First, a theoretical implication of these findings is that our serial mediation model provides strong evidence for the ABC theory of emotions. That is, our results revealed the effect of physical status on emotions and self-perceived quality of life mostly via cognition for older adults in China. Moreover, a practical implication is that emotion regulation training may be useful to improve the quality of life. Among these training and strategies, cognitive reappraisal may be more useful for older individuals. Another important practical implication is that older individuals in China value physical health more than mental health. Maintaining and improving physical health is a priority in order to improve the quality of life of older people in China.

Although there are some strengths and implications, several limitations need to be noted. First, because we utilized cross-sectional data from the wave of 2017–2018 in CLHLS, the causal sequence of variables cannot be determined. Specifically, although our hypotheses were established on the basis of theories, the cross-sectional nature of the current study cannot determine temporal association. Second, potential pitfalls of the self-report measurement need to be considered when interpreting our results. For example, the self-report data are subject to a social desirability bias. The linguistic comprehension of older individuals may also affect the self-report data. Finally, the present study investigated the effect of physical health on self-perceived quality of life among older adults in China, while other important influential factors of quality of life (e.g., interpersonal relations) have not been fully examined in the present study.

## 6. Conclusions

In conclusion, the present study examined the effect of physical health on self-perceived quality of life among older individuals in China. The effect size of physical health was small to moderate but was larger than that of negative emotions. Moreover, the positive cognition and negative emotions independently and serially mediated the association between physical health and self-perceived quality of life. These findings may contribute to researchers getting a better understanding of the ABC theory of emotion. Furthermore, the findings offer some insight into the influential factors of self-perceived quality of life among older individuals in China. Except for physical health, positive cognition, and mental health, there are probably some important and unique influential factors for quality of life for older people in the context of Chinese culture.

## Figures and Tables

**Table 1:** Descriptive statistics of the participants.

Variable (N = 15,874)	Number (Percent)/Mean ± SD
Age (years)	85.46 ± 11.70
Gender (female: male)	8949 (56.4%): 6925 (43.6%)
Marital status	
Currently married and living with spouse	6135 (38.6%)
Separated	276 (1.7%)
Divorced	52 (0.3%)
Widowed	9004 (56.7%)
Never married	140 (0.9%)
Other	267 (1.8%)
Total number of cases	15,874

**Table 2:** Correlation analysis of all continuous variables.

Variable	Mean	SD	1	2	3
1. Physical health	2.57	0.86	--		
2. Positive cognition	2.08	0.68	0.370 **	--	
3. Negative emotions	7.98	1.44	−0.308 **	−0.409 **	--
4. Quality of life	2.11	0.76	0.496 **	0.379 **	−0.278 **

*Note.* N = 15,874. ** *p* < 0.01.

**Table 3:** Standardized coefficients for total and direct effects on positive cognition, negative emotions, and quality of life in the serial mediation model.

Variable	Positive Cognition	Negative Emotions		Quality of Life	
	Direct Effect	Total Effect	Direct Effect	Total Effect	Direct Effect
Physical health	0.370 ***	−0.308 ***	−0.182 ***	0.497 ***	0.400 ***
Positive cognition			−0.342 ***	0.226 ***	0.202 ***
Negative emotions					−0.072 ***
R^2^	0.137	0.196		0.295	

*Note.* N = 15,874. *** *p* < 0.001.

**Table 4:** Total, individual, and serial indirect effects for physical health on quality of life and bias-corrected 95% confidence intervals.

Pathway	Indirect Effect	SE	Bias-Corrected 95%CI	
			Lower	Upper
Total indirect	0.097	0.004	0.089	0.105
Physical health→Positive cognition→Quality of life	0.075	0.004	0.067	0.082
Physical health→Negative emotions→Quality of life	0.013	0.002	0.010	0.016
Physical health→Positive cognition→Negative emotions→Quality of life	0.009	0.001	0.007	0.011

*Note.* CI = confidence interval.

## Data Availability

The data presented in this study are available on the Peking University Open Research Data Platform (https://opendata.pku.edu.cn/ accessed on 24 June 2021). Some variables are restricted to preserve the anonymity of study participants.
